# Does financial development and renewable energy consumption impact on environmental quality: A new look at China’s economy

**DOI:** 10.3389/fpsyg.2022.905270

**Published:** 2022-10-14

**Authors:** Qiang Fu, Junwei Wang, Yonghui Xiang, Samina Yasmeen, Bojun Zou

**Affiliations:** ^1^School of Economics, Shandong University, Jinan, China; ^2^School of Media and Law, Ningbo Tech University, Ningbo, China; ^3^School of Economics and Management, Zhejiang University of Science and Technology, Hangzhou, China; ^4^Government Special Education Center Daultala, Rawalpindi, Pakistan; ^5^School of International Education, Changchun Institute of Technology, Changchun, China

**Keywords:** global warming, renewable energy projects, NARDL model, financial development, environmental quality

## Abstract

Environmental problems such as climate change have brought to light the necessity of implementing more stringent environmental regulations and expanding the use of renewable energy sources in order to protect the environment and maintain a green ecosystem. As a result, this study aims to investigate the impact of China’s financial development and consumption of renewable energy on the country’s environmental quality from 2009 to 2019. Following the application of the ARDL method, this research begins by employing the NARDL (non-linear autoregressive distributive lag) model in order to analyze the asymmetry in the data that results from the presence of either positive or negative aspects of financial development. The results of the NARDL bound test indicate that the variables are long-term co-integrated. This enables the application of the ARDL methodology. The ARDL bound test findings show a positive relationship that exists over the long-term between financial development, trade openness, renewable energy consumption, economic growth, and CO_2_ emissions. In addition, the error correction model (ECM) provides evidence that there is, at least in the short run, a connection between CO_2_ emissions, financial development, economic growth, and energy consumption. Furthermore, according to a dynamic multiplier graph, the positive aspect of financial development has a greater influence on carbon emissions for a longer time than the shocks associated with a less favorable financial development. According to the findings, there does not appear to be any asymmetry between CO_2_ emissions and financial development, which supports the idea that both the positive and negative aspects of financial development have an equally significant impact.

## Introduction

The increasing environmental danger presented by economic growth and development measures and activities is a fiercely discussed study topic in environmental economics and sustainable development (Wu et al., 2019; Lv et al., 2021). Thus, financial growth and environmental preservation are two of the most pressing challenges facing today’s global world. They have moved to the top of national and international agendas in developing and developed economies (Wu et al., 2020, 2021). An increasing number of economists and environmentalists worldwide are interested in finding out how economic expansion relates to improvements in environmental quality (Chen et al., 2021; Wu and Zhu, 2021). Besides boosting the efficiency of the financial system, a well-functioning finance system has a substantial effect on the economy’s overall performance.

During the late twentieth century, when the potential costs of increased financial growth began to spook people in the form of negative externalizes and accelerated their growth, the term “climate change” was created by (Chang, 2014) in replacement of the word “global warming.” As a result of the concerns expressed by both industrialized and developing countries about the consequences of CO_2_ emissions on global warming, environmental conservation has emerged as a universal priority (Lei et al., 2021; Pan et al., 2021). Even though investigators have found a connection between economic expansion and CO_2_ emissions (Al-Mulali et al., 2015; Fagbemi, 2021; Tang et al., 2021), the findings for developed and developing countries have come to very different conclusions. It has been shown that increased financial development can reduce CO_2_ emissions, for example, by conserving the environment (Le et al., 2020; Kong and Gallagher, 2021; Lv and Li, 2021a). Several studies have demonstrated that financial development causes environmental degradation (Xiao et al., 2020; Qiao et al., 2022). In addition to this research, a few others (Deng and Zhao, 2022) have concluded no significant association between financial development and CO_2_ emissions. This is contrary to Nyoka (2019) findings, who report inconsistent findings in this area. According to the findings of this study, financial development can assist businesses in overcoming planning constraints and accomplishing scale economies in the production process, which will ultimately result in less carbon emissions as a result of the reduction of waste.

Next, from now until 2020, China has set aims to utilize renewable energy sources. Considering the total production capacity and the anticipated contribution of renewable energy to total power generation, these goals are significant in terms of overall installed capacity and energy production (Chai et al., 2021; Yang et al., 2021b; Ren et al., 2022). One of the most important goals of China’s renewable energy development is to reduce CO_2_ emissions and its dependence on foreign energy by isolating rising fossil energy consumption from economic growth over the next several decades (Zhou et al., 2020). This decoupling is also expected to positively impact the air and water quality in the surrounding area. Between 1995 and 2003, it was estimated that environmental pollution cost the country more than 4% of its gross domestic product (Moşteanu, 2020). Focusing on renewable energy is also intended to increase China’s ability to compete as the largest multinational distributor of clean, low-cost renewable energy technologies currently in short supply (Wu and Zhu, 2021; Wang and Luo, 2022; Zhang et al., 2022). Specifically, we measure the effects of China’s renewable energy targets on the utilization of renewable and fossil energy sources and the impact on CO_2_ emissions, which are critical to legislators in China and around the world.

As discussed above, renewable energy consumption and financial development can play an important role in controlling environmental quality. Before we move to the contribution section, it is necessary to introduce how renewable energy consumption and financial development are linked with environmental quality. This study investigates the connection of financial development with environmental quality because it is uncertain in the selected region for the FD-environment nexus from 2009 to 2019. Numerous case studies have tried to explore this connection, but they proposed different logic or opinions and have not reached a single point; therefore, this study attempts to examine this connection before suggesting any policy. However, from the perspective of the theoretical impact of financial development on carbon emissions, academia has proposed various schools. According to the research of some scholars (Grohmann et al., 2018; Liu et al., 2021; Usman et al., 2022), the expansion of the financial sector helps to reduce carbon emissions for the following reasons: (1) To reduce production costs and improve the market competitiveness of products, companies must regularly update their production technology and equipment, which requires sufficient financial support. (2) In order to cope with environmental deterioration, the government usually initiates various environmental protection projects, promotes the overall industrial transformation, and encourages the use of clean energy. A developed financial system can make it easier for companies to do these jobs by effectively easing financing constraints, further reducing energy costs and carbon emissions. Financial institutions can provide necessary funds for these projects or plans according to corresponding policy arrangements, which will help improve energy infrastructure and ultimately reduce carbon emissions. (3) NARDL econometric technique is applied in the current study to measure the environment’s quality. NARDL provides an efficient method for capturing the asymmetry component of China’s financial development and CO_2_ emissions. Moreover, it is critical to examine the asymmetry effect in order to determine whether the negative or positive component dominates China’s CO_2_ emissions. Additionally, this study will look for any asymmetry in the relationship between financial development and CO_2_ emissions; if any is found, it will be utilized in place of previous studies that assumed the relationship was symmetrical. Asymmetrical financial development will examine long-and short-term linkages using the ARDL approach.

The remaining portions of the paper are laid out as follows: The literature review is discussed in section “Literature review,” the data and methodology of the study are analyzed in section “Methodology and data sources,” and the empirical findings, as well as a conclusion and some policy recommendations, are presented in section “Results and discussion” and section “Conclusion and policy implications,” respectively.

## Literature review

### Financial development and environmental quality

As described in the section “Introduction,” a new study topic has been formed that uses a recently developed carbon emissions monitoring system-based on consumption (Fang et al., 2022). The connection between CO_2_ emission and various other economic and social factors remains largely unexplained despite decades of research. From 1990 to 2008, Ali et al. (2021) examined how countries with high earnings fared in relationships between CO_2_ emission and financial development. Their study was one of the first to be published in this field. They looked into how the rise in financial development affected the amount of CO_2_ emissions in territorial and consumption development (Probst et al., 2021). Growth has a favorable impact on territorial and carbon emissions; however, the effect on carbon emissions is greater than on territorial carbon emissions, as seen in the graph below. Xu et al. (2017) studied 14 major economies between 1995 and 2009, taking into account various factors. The researchers considered carbon emissions in production and consumption contexts when conducting their investigation. The data demonstrate that, while financial development and both forms of carbon emissions have a constructive association, consumption-based carbon emissions have a more positive link than other carbon emissions.

As a result of the increasing importance of environmental issues, academics and politicians emphasized economic growth. Everybody fundamentally criticizes the states’ potential expansion because growth entails increased use of natural resources and development, both of which have a negative environmental impact in the form of CO_2_ emissions, which everyone despises (Hao et al., 2021; Umar et al., 2021; Wang et al., 2021). Studies in this area have yielded mixed results, which is not surprising. A few studies suggest that growth has a bidirectional causal relationship with carbon emissions. In contrast, others say that growth has the opposite effect, and expansion reduces CO_2_ emissions. For example, Huang et al. (2021) discovered that income and CO_2_ emissions are correlated in both directions. According to (Abokyi et al., 2019; Charfeddine and Kahia, 2019), financial development and carbon emissions are bidirectional. Both studies were done between 1990 and 2011, the most recent being in 2011. Similarly, Lee et al. (2022) investigated the impact of financial development on environmental quality in the income groups over the period of 1990–2017. They employed the AMG estimator and found the significant role of financial development in environmental reduction only for high-income economies. With data spanning 1990–2017, Cheng and Degryse (2010) looked at the relationship between financial development and macroeconomic indicators, including carbon emissions. According to their findings, as the economy grows, so do carbon emissions, which they attribute to human activity.

Researchers have examined the correlation between economic growth and power consumption over the last few years, and their findings have been pooled (Liu et al., 2022; Zhang et al., 2022). On the other hand, some studies have found that growing financial development increases energy utilization. For instance, the World Bank investigated the impact of monetary expansion on energy consumption in 23 rising nations (Ma et al., 2022). Abdullah et al. (2021) demonstrated the role of financial development in renewable energy consumption in United States economy. They employed the Fourier Quintile regression and pointed out at higher quintile it cause of renewable energy consumption. Among the methods employed by the author were a linear dynamic panel model and a generalized methodology of moments. The empirical research demonstrates that financial growth positively and significantly impacts energy utilization. A study using the ordinary least squares (OLS) technique (Ghosh and Vinod, 2017) showed that financial gain has a significant favorable effect on energy utilization in China. The impact of financial inclusion, financial development, and expenses of energy that are modified according to the season for energy consumption and CO_2_ emissions will be investigated using NARDL techniques to determine the influence of renewable energy consumption.

### Renewable energy and environmental quality

For the past two decades, one of the most impactful ways to combat the rise in greenhouse gas emissions worldwide has been to switch from energy generated by fossil fuels to energy derived from clean, renewable sources (GHGs). Renewable energy improvements in quantity and efficiency are already being considered in GHG reduction policies at different organizations. Njindan Iyke and Odhiambo (2017) analyzed the United States from 1990 to 2015, focusing on the influence that CO_2_ emissions are reduced by natural gas and renewable energy. Their findings demonstrate that, over the long-term, using energy that comes from renewable sources results in a sizeable drop in CO_2_ emissions. Li et al. (2020). conducted a study on the BRIC countries from 1985 to 2016 within the context of the Economic Knowledge Consortium. The investigation into the connection between CO_2_ emissions, non-traditional sources of energy, and natural gas exposes a poor association between CO_2_ emission and natural gas and a negative association between renewable energy and carbon emissions in such nations. Natural gas is also shown to have a weak relation with CO_2_ emission. Likewise, a case study of selected Asian economies by Shah et al. (2020) tried to investigate the role of renewable energy consumption to environmental quality over the period of 1990–2017. By employing the CCE-MG they found the positive contribution of RE (biomass) to environmental damages.

Moreover, Vo et al. (2021) 128 nations were studied from 1990 to 2014, and the impact of renewable energy sources on carbon emissions was analyzed. They found that nations with a high % age of renewable energy in their power mix improve the environment to a more significant level than that of other nations. This finding was based on the observation that countries with more considerable % age of renewable energy in their energy supply prevent climate change. Likewise, another case study of WANA economies by Shah et al. (2020) investigated the role of renewable energy consumption in environmental quality and they found the inverse association between RE and environmental degradation. Zaidi et al. (2019) conducted a study of 30 nations spanning the period between 1989 and 2018, during which their energy supply has both nuclear energy and renewable energy. Their results demonstrate that the impact of renewable energy on CO_2_ emissions decrease is significantly more significant than the impact of atomic energy. Lee et al. (2022) also tried to investigate the role of renewable energy consumption in different income group. They found the negative relationship between the RE and carbon emissions only for higher and upper middle income countries. The EKC paradigm (Samour et al., 2022) examined the connection between renewable energy sources and the number of carbon emissions produced in 66 emerging regions from 1987 to 2018, using data from 1990 to 2014. Energy efficiency and the usage of renewable energy, according to their research, have a major impact on carbon emissions reduction. According to (Zaidi et al., 2021), renewable energy has a negative effect on global carbon emissions. A study conducted by the World Resources Institute examined 120 nations from 1995 to 2015 and discovered that renewable energy cuts carbon emissions worldwide, although only somewhat. This discovery demonstrates that fossil fuel energy consumption is increasing significantly quicker than the consumption of renewable energy in general (Liang and Reichert, 2012). However, the influence of renewable energy on carbon emissions reductions varies depending on the income level of a country’s subgroup of countries.

There have been few studies that have taken into account CO_2_ emission consumption level as opposed to renewable sources of energy. Sheraz et al. (2022) conducted the first research, which concluded that there are two ways to reduce CO_2_ emissions-based on consumption. To lower total consumption of products, which will lead to a lower level of intake of CO_2_ emission encapsulated in those services and goods, one must first lower total consumption of services and goods. This will lead to a significant reduction of consumption-based CO_2_ emissions enshrined in those services and goods. The following method is used to reduce carbon emission possessions, thereby lowering the intensity of emissions throughout the manufacturing process (Omri et al., 2015). In the recent time, Zhang et al. (2022) tried to discuss the production side of green energies and they found hydro and geothermal energies cause of environmental damages. To achieve the other, many companies should switch to using renewable energy sources rather than fuels derived from fossil fuels, particularly in the power production industry, all over the world. According to their investigation into the relationship between energy productivity and eco-innovation and consumption-driven CO_2_ emissions, Martí-Ballester (2021) also considered consumption-based CO_2_ emissions. It was built using data from G7 countries from 1990 to 2018, with renewable energy included as a variable in the model. Their findings demonstrate that the usage of renewable energy reduces carbon emissions resulting from consumption. Zhang et al. (2022) also tried to investigate the role of green energies in sustainable growth for the great 10 economies. Concluding remarks showed that biomass and hydro energy consumption cause to boost the level of sustainable growth. Besides, Shah et al. (2020) also investigated the impact of energy consumption on environmental quality and they found the supportive role of energy in environmental quality. A case study of top tourist economies in Asia Shah et al. (2020) elaborated the significant role of renewable energy consumption in emissions reduction.

There may rise a think how our research adds to the previous body of literature? In conclusion, the previous studies appear to be lacking in their selection of the role of the financial and energy sector energies, and they failed to take into account how critically important this role is in determining the quality of the environment for China. The primary objective of this study is to provide an assessment of the core of the issue that is becoming more widespread around the world, in particular in developing economies such as China. As a result, in order to fill this gap, the study has concentrated on renewable energy as well as the financial sector, both of which may cause issues with the environment. In response to these constraints, we take into account a variety of additional factors and use an appealing model to investigate how those factors are related to environmental deterioration in a more precise and comprehensive manner. The findings shed new light on the topic, and as a result, both the empirical and the theoretical bodies of research will benefit from this study. In addition, in order to eliminate the inconsistencies that are present in the literature, the current study makes a contribution to the literature by re-investigating the role of economic growth and trade openness in environment quality in selected economies between 2009 and 2019. In addition to this, the results of the non-autoregressive distributive lag model (N-ARDL) will provide proper policy suggestions for a chosen economy in order to achieve low environmental degradation and deal with consumption side problems as well as desired environmental goals.

## Methodology and data sources

### Model specification

The NARDL model predicted the usage of renewable energy utilization and the development of financial resources. When applied to time series of varying integration levels, NARDL is a model that can serve a variety of purposes and can manage potential relationships linking time series with varying degrees of inclusion involving vector error checking (Li et al., 2021; Yang et al., 2021a). The mixture of I(0) and I(1) time series is indeed possible with this method of calculation 1Traditional multiple regression models, like those proposed by Jiang and Ma (2019) and Umar et al. (2020) and others, all demand time-series synchronization in addition to getting an I = 1 result. The internal and external factors are co-linear in relation to the independent and dependent variables; they are no longer applicable and may result in incorrect interpretation of the conclusions. While the NARDL model is useful in this scenario, it is not appropriate in other temporal series that are not more linked, although their long-term information is attached. When analyzing the performance of economic and energy policy initiatives, it is critical to look for hidden multi-co-linearity as a trend (Falcone et al., 2018). Even more crucially, The NARDL method is sufficiently straightforward to eliminate the possibility of under-or over-fitting occurring in mathematical methods in either the long-or short-term. During both the short and long simulation process, it considers the intermediation deformation between the independent factors. According to (Ozturk and Acaravci, 2013), energy plans that are successful and trustworthy can only be developed with precise projections.

Non-linear autoregressive distributive lag model studies non-linearities in general, and (Dafermos et al., 2021) used the Brock–Decherd–Steinman (BDS) time-varying test, as well as a test of both independent and dependent variables-based on the ICSS’s most recent data of (Baloch et al., 2021), to shed light on the difficulties contained within time-series commodities set of data. When using linear regression, there are two layers to consider: one in the dynamics of period returns and the other from the organization of conventional statistical decisions concerning how to deal with return correlations. First and discoveries must be made. The following illustrates the ARDL: n-stationarities are required to capture both short-and long-term imbalanced consequences, and this number is sufficient. It is now widely recognized that numerous parametric variables depict market and business arrangements. For example, Chams and García-Blandón (2019) employed the BDS test to identify the presence of non-linearity in stock market data, which they found to be true.


(1) $$\begin{array}{c} \begin{array}{l} \Delta ln{\mathrm{CO}}_{2_{t}}=\alpha_{0}+\alpha_{1}\ln{\mathrm{CO}}_{2_{t-1}}+\alpha_{2}{\mathrm{lnFD}}_{t-1}\\ +\alpha_{3}\ln{\mathrm{GDP}}_{t-1}+\alpha_{4}{\mathrm{lnPOP}}_{t-1}+\alpha_{5}{\mathrm{lnTO}}_{t-1}+ \end{array}\\ \begin{array}{l} \overset{p-1}{\underset{i=1}{\sum }}\delta_{i}\Delta {\mathrm{lnREC}}_{t-i}+\overset{q-1}{\underset{i=0}{\sum }}\gamma_{i}\Delta {\mathrm{lnFD}}_{t-i}+\overset{q-1}{\underset{i=0}{\sum }}\omega_{i}\Delta ln{\mathrm{GDP}}_{t-i}\\ +\overset{q-1}{\underset{i=0}{\sum }}\phi_{i}\Delta {\mathrm{lnPOP}}_{t-i}+\overset{q-1}{\underset{i=0}{\sum }}\pi_{i}\Delta {\mathrm{lnTO}}_{t-i}+\varepsilon_{t} \end{array} \end{array}$$

The use of equation-1 over traditional co-integration processes has a number of advantages; however, in a context where fact drives the connection among economic and financial factors to change, they may become too basic and retrogressive. The non-linear generalization of the regular regression analysis provided by Equation (1) results in the NARDL model:


(2) $$\begin{array}{c} \begin{array}{l} \Delta ln{\mathrm{CO}}_{2_{t}}=\alpha_{0}+\alpha_{1}\ln{\mathrm{CO}}_{2_{t-1}}+\alpha_{2}^{+}{\mathrm{lnFD}}_{t-1}^{+}\\ +\alpha_{3}^{-}{\mathrm{lnFD}}_{t-1}^{-}+\alpha_{4}^{+}\ln{\mathrm{GDP}}_{t-1}^{+}+\alpha_{5}^{-}\ln{\mathrm{GDP}}_{t-1}^{+} \end{array}\\ +\alpha_{6}^{+}\mathrm{lnPO}P_{t-1}^{+}+\alpha_{7}^{-}\mathrm{lnPO}P_{t-1}^{-}+\alpha_{8}^{+}{\mathrm{lnTO}}_{t-1}^{+}+\alpha_{9}^{-}{\mathrm{lnTO}}_{t-1}^{-}+\\ \begin{array}{l} \overset{p-1}{\underset{i=1}{\sum }}\delta_{i}\Delta {\mathrm{lnREE}}_{t-i}+\overset{q-1}{\underset{i=0}{\sum }}\left(\gamma_{i}^{+}\Delta {\mathrm{lnFD}}_{t-i}^{+}+\gamma_{i}^{-}\Delta {\mathrm{lnFD}}_{t-i}^{-}\right)\\ +\overset{q-1}{\underset{i=0}{\sum }}\left(\omega_{Y}^{+}\Delta ln{\mathrm{GDP}}_{t-i}^{+}+\omega_{Y}^{-}\Delta ln{\mathrm{GDP}}_{t-i}^{-}\right)+ \end{array}\\ \begin{array}{l} \overset{q-1}{\underset{i=0}{\sum }}\left(\phi_{\mathrm{ROP}}^{+}\Delta \mathrm{lnPO}P_{t-i}^{+}+\phi_{\mathrm{ROP}}^{-}\Delta \mathrm{lnPO}P_{t-i}^{-}\right)\\ +\overset{q-1}{\underset{i=0}{\sum }}\left(\pi_{\mathrm{TO}}^{+}\Delta {\mathrm{lnTO}}_{t-i}^{+}+\pi_{\mathrm{TO}}^{-}\Delta {\mathrm{lnTO}}_{t-i}^{-}\right)+\varepsilon_{t} \end{array} \end{array}$$

(3) $${\mathrm{lnFD}}_{t}^{+}=\overset{t}{\underset{j=1}{\sum }}\Delta {\mathrm{lnFD}}_{j}^{+}=\overset{t}{\underset{j=1}{\sum }}\max\left(0,\Delta {\mathrm{lnFD}}_{j}\right)$$

(4) $${\mathrm{lnFD}}_{t}^{-}=\overset{t}{\underset{j=1}{\sum }}\Delta {\mathrm{lnFD}}_{j}^{-}=\overset{t}{\underset{j=1}{\sum }}\min\left(0,\Delta {\mathrm{lnFD}}_{j}\right)$$

NARDL’s short-and long-term asymmetrical effects on renewable energy consumption must be tested to understand the model properly. An ARDL of the null *i* = 1, 2, …, *q* − 1 is used in this research to describe the brief improper impacts of the variables of macroeconomics on the utilization of renewable energy, while a regression model is used to analyze the long-term non-linear impact on the consumption of renewable energy by the financial development. *i* = 1, 2, …, *q* − 1 and *i* = 1, 2, …, *q* − 1, in which particularly related explanatory variables are expected are used. As suggested by the expression, an over-parameterized variance decomposition structure may result if the normal test fails to eliminate the assumption of prolonged or fairly short imperfect information for at minimum one explained variable (Liu and Xiao, 2018). A single increment in potential drivers can be used to observe the impact of that boost on renewable energy consumption using static factors. It has two different effects on renewable energy production, which is commonly classified into two categories: a one-unit gain in the stock exchange, Financial development’s long-term impact on renewable energy consumption is analyzed to use an ARDL, where _FD + =−FD+/REC is used to estimate the descriptive variables that are related with the null _FD+, while the _FD-is used to predict the descriptive variables that are related with the _FD+. As the equation suggests, the over variance decomposition structure means that normal tests may fail to exclude certain suppositions of asymmetric information for at least one descriptive variable. Analyzing how a single growth in determinant factors impacts renewable energy usage can be done using static variables. When the stock market rises by one unit, it has a two-fold influence on renewable energy needs:


(5) $$m_{h}^{+}=\overset{h}{\underset{j=0}{\sum }}\frac{\partial REC_{t+1}}{\partial FD_{t}^{+}}\mathrm{and}m_{h}^{+}=\overset{h}{\underset{j=0}{\sum }}\frac{\partial REC_{t+1}}{\partial FD_{t}^{+}}$$

We can see how the original equivalency and subsequent change are affected by a disturbance to the investigated variable path by employing extremely non-linear integrators (s). As the NARDL model specification is dependent on the adjustment route structure, it underlines the need to appropriately characterize the random effect model before conducting an analysis of the collection of variables under examination.

### Data sources

Data-driven empirical research from 2000 to 2019 was conducted in 30 Chinese provinces. These numbers were culled from various sources, including the Statistics Yearbook of urban China and the websites of several provinces and municipal agencies. To exclude the impact of pricing, GDP, and FDI were artificially inflated to 2007 as a baseline. Missing values were also filled in using interpolation methods.

### Variable description

A first evaluation of the components’ performance was made using descriptive and inferential statistics. Table 1 shows the results of each equipment unit regression analysis. Testing the data for functional underlying causes utilized parallel and cultural changes. All but three investigations yielded a unit root test in the quantity of renewable energy consumed. This proves that renewable energy usage is at a steady level. The (Weili et al., 2022) and Fisher augmented dickey fuller unit root tests provide further evidence of a standstill in financial advancement. However, energy costs were shown to be adequately positioned at GDP’s dependent and independent variables, despite GDP’s non-stationary nature. After a single change, all variables remained stable (Song et al., 2021). Therefore, it was deemed vital to determine whether the factors were linked over an extended period of time. Results from the correlation analysis are shown in Table 1. Growing financial and monetary resources influence renewable energy use significantly, but the energy cost has minimal effect. The rise of the financial sector helps promote the usage of renewable energy. A 0.07% rise in renewable energy use is associated with each 1% gain in financial development. In some places in China, economic expansion has a substantial impact on the usage of wind resources.

**Table 1:** Descriptive statistics.

	lnCO_2_	lnFD	lnGDP	lnPOP	lnREE	lnTO
Mean	0.799	0.691	6.782	4.739	2.213	3.041
Maximum	2.073	0.733	8.963	5.000	2.246	4.166
Minimum	−0.555	0.791	4.883	4.261	2.566	1.593
Std. Dev.	0.784	0.086	1.254	0.216	1.825	0.817
Skewness	0.047	0.509	0.234	−0.655	0.220	0.407
Kurtosis	2.053	0.107	1.731	2.264	1.525	1.713
Jarque–Bera	2.149	4.474	4.346	5.363	0.466	5.503

## Results and discussion

### Unit root, CD, and co-integration

The results of the unit root test are performed first in order to determine the co-integration in time series data. The unit root test was applied before the ARDL bound test. Stationarity issues can be resolved using unit root tests, and there is no integration of any variables at the I level (2). The empirical data outcomes at the level and first difference were used in the Augmented Dickey-Fuller test (Ling et al., 2021; Ohajionu et al., 2022). The findings contradict the null hypothesis by showing that CO_2_ emissions, financial development, population, economic growth, renewable energy, and trade openness are all stationary at the 5% level of significance I(0) and I(I) (see Table 2).

**Table 2:** Panel unit root tests.

ADF	*t*-statistics	*p*-value	*t*-statistics	*p*-value
lnCO_2_	−0.5959	0.8628	−5.4315	0.0000***
lnFD	0.7289	0.9918	−4.7688	0.0003***
lnGDP	1.8768	0.9998	−3.8884	0.0039***
lnPOP	−3.3610	0.0170**	−0.8979	0.7815
LnREE	−2.7721	0.0689*	−5.8710	0.0000***
lnTO	−1.1549	0.6876	−5.1780	0.0001***

***, **, and * shows the level of significance at 1%, 5%, and 10%.

The co-integration relationship between Gross Domestic Product, Population Density, Industrialization, Trade Openness, and carbon emissions is determined in Table 3. The F-statistic calculated shows that the observed value is greater than the upper bound critical value at a significance level of 0.05%. We can conclude that the null hypothesis is not true because the F-statistic (5.202) is greater than the upper bound value. Consequently, co-integration among the variables I(1) and I(0), and the assumption that the variables are linked for at least some period of time in either the short run or the long run (Shah et al., 2020).

**Table 3:** ARDL bounds test for co-integration.

Significant if	I(0) (lower bound)	I(1) (upper bound)
1%	2.046	2.808
2.50%	1.950	2.694
5%	1.572	2.274
10%	1.356	2.010
*F*-statistics	5.202	

### Results of the NARDL model

Table 4 contains the estimated values of the NARDL coefficients for both the short run and the long run. According to the results of the short-run analysis, the estimates are positive not only for FD increases but also for FD drops. For example, the projected elasticities of FD rise (drop) affecting CO_2_ emissions are 0.097 (0.045), which indicates that a 1% expansion (contraction) in FD is projected to rise (drop) carbon emissions by 0.033%. Similarly, the estimated elasticities of FD rise (drop) by (0.0334%) concerning to FD are 0.097 (0.045). The coefficient of FD rise is greatly significant from a statistical point of view, whereas the coefficient of FD decrease is not significant. The distinction in FD appears to have an asymmetric influence on China’s carbon emissions in the short run, based on the various statistical significance outcomes and the path of the processed elasticities. This is the case even in the longer term. The Wald test suggests that the alternative hypothesis of short-term symmetry should be acknowledged at the 1% level. This suggests a robust indication of the asymmetric results of FD variations on short-run outcomes. On the other hand, the findings indicate that both estimates are positive when viewed over a longer period of time. For example, the elasticity of FD rises (drops) concerning CO_2_ emissions is 0.097 (0.046), which indicates that a 1% increase (drop) in FD is projected to rise (drop) CO_2_ emissions by 0.401%. Similarly, the elasticity of FD drops (rises) concerning CO_2_ emissions is 0.036 (0.036%). Only in the event that FD rises are observed in the short term does it produce a statistically significant deviation from zero. This provides a sturdy indication of the long-term symmetric results of FD variations. In Equation 3, both the short-run and long-run estimates have the potential to be significant, provided that the variables in the model are associated with one another through a co-integration relationship. Therefore, before moving on to the next section, it is important to conduct a test to determine whether or not there is co-integration between financial development, energy consumption, CO_2_ emissions and economic growth. In one variation of this test, the processed F-statistic is compared to the upper acute value arranged by Feng and Wu (2022). This is just one of several possible approaches. When this method of operation is carried out, the F-statistic is estimated. As a result, the hypothesis of long-run co-integration between variables is widely acknowledged as accurate.

**Table 4:** Non-linear autoregressive distributed-lag (NARDL) equation results.

Variable	Short-run coefficient	SE	*t*-statistic
FD^+^	0.097***	0.052	2.338
FD^−^	0.046**	0.130	0.437
ΔRE	−0.856***	0.144	−7.433
ΔREE (−1)	−0.529***	0.146	−4.535
ΔGDP	0.050***	0.015	4.237
ΔTO	0.316**	0.097	3.796
ECT (−1)	−0.313***	0.104	−3.755
**Variable**	**Long-run coefficient**	**SE**	***t*-statistic**
FD+	0.386***	0.136	3.539
FD	0.182*	0.543	0.418
REE	−1.302***	0.166	−9.777
GDP	0.200***	0.088	2.837
TO	0.517	0.233	4.143
Constant	9.252	1.127	10.259
Diagnostic test			
*R* ^2^	0.974		
F-statistics	1546.268		
Prob(F-statistics)	0.000		
DW	1.630		
	0.000		
LM	3.964		
	(1.256)		
WaldLR	11.057		
	[0.0054]		
WaldSR	78.748		
	[0.000]		
*ARCH*	23.979		
	(1.466)		

***, **, and * shows the level of significance at 1%, 5%, and 10%.

In order to conduct a reliable analysis, we make use of the diagnostic test listed at the very bottom of Table 4. The findings suggest that both the serial correlation LM tests and the heteroscedasticity are higher than the level of 5% in this model; consequently, we accept the hypothesis that the null value exists. The most important thing is the ECM (error correction model) in the short run. The outcomes express that the aggregation in the model is 0.46% speed of amendment, which is also significant (0.0000), and this is the ideal situation. We carried out a series of analytic tests both in the short and long-run tables to establish a circuitously long-run model relationship. Since the environmental Kuznets curve (EKC) is a phenomenon that occurs over the long run, it is possible to justify its existence in the short run. The results of the problem-solving test show those above. The value of r-squared specifies that a self-regulating variable accounting for 88% of the total variation is connected to trade openness, population density, and when attempting to assess economic growth.

According to (Dogan and Seker, 2016), a small movement in the renewable energy business could significantly impact sustainability. According to Zhuo and Qamruzzaman (2022), an alternative to coal has the potential to be utilized to create power and provide a low-cost source of energy. Some regions are still largely reliant and other fossil fuels as their principal means of generating electricity, while others do not. On the other side, even though renewable energy sources have expanded substantially around the globe in recent years, % age of total energy utilization continues to be insufficient. This is because electricity generation in developed countries is expanding moderately, making it difficult to change existing infrastructure or consumer habits. Despite increased energy demand, fossil fuels continue to be essential in developing countries. Alternative energy sources cannot compete with fossil fuels such as coal and petroleum in economics. As a result (Mujahid and Akhtar, 2014), it is projected that increased renewable energy use will take some time to meet the energy demand. Despite this, national authorities are working to improve renewable energy consumption and develop new renewable energy technologies (Fu et al., 2021; Mohsin et al., 2021; Feng et al., 2022).

According to (Ahmad et al., 2019), 14% of the total energy demand in the world is met by renewable energy sources. Biomass, hydropower, geothermal, solar, wind, and marine energies are all examples of renewable energy sources. The development of systems that use renewable energy will make it possible to solve the current most important problems, such as increasing the dependability of energy supply and enhancing fuel efficiency. When looking at numerous different model scenarios, there is reason to assume that microfinance institutions and the general financial and economic growth council positively impact renewable energy consumption. Interestingly, in their research on Bangladesh (Yang and Ni, 2022), discovered the same thing we did in our current study, which is noteworthy. Only the model choice that is the most inclusive has the potential to have a beneficial effect on the value of this factor whenever it relates to the availability of financial companies.

In contrast to prior research findings, in none of the models shown in Table 4, the coefficients of another functional capability, like those of lending enterprises and the financial industry, are positive or statically relevant, although they were in previous studies. When it comes to the efficacy of monetary policy and financial institutions, there is no feasible difference between developed and developing countries regarding the awareness of viable energy (Sheraz et al., 2022). The findings also demonstrate that the demand for renewable energy in these state governments is highly driven by changes in currency rates, interest rates, and government spending. Table 5 further shows that the influence of complete financial development on the usage of other forms of energy is minimal because this factor contributes a minor amount to model replacements, consistent with previous findings.

**Table 5:** Granger causality tests.

Excluded	Chi-square	df	Prob.
LnRE	0.8974	1.05	0.433
LnFD	15.357	1.05	0.000
lnEQ	14.998	1.05	0.000
lnCO_2_	10.066	1.05	0.003
lnROP	14.967	1.05	0.000
lnTO	3.0002	1.05	0.279
lnBI	3.6523	1.05	0.089
All	60.747	9.45	0.000

According to the findings from ARDL, the level of accessibility that bankers have to governmental entities, the breadth of financial firms’ offerings, the effectiveness of state agencies, and their linkages with financial markets are all found to have significant correlations with renewable energy production. Following the publication of these findings, it is anticipated that reforms in these institutions and markets will significantly impact the electricity generation sector in EU member states (Hou et al., 2019; Hsu et al., 2021; Yumei et al., 2021). In the short term, real oil prices impact the utilization of renewable energy. The effect of actual product pricing on consumers’ demands for renewable energy sources is negligible. When the oil price goes down, alternative forms of energy have a beneficial and noticeable influence on the rest Financial Development Index, even though oil prices are falling.

However, models which include domestic production dependent on banks and stock exchanges have a detrimental impact on the economy in the short term, even though good gains in economic growth have an excellent effect. The use of renewable energy sources, on the other hand, is positively influenced by environmental and industrial prosperity. In contrast (Charfeddine and Kahia, 2019), it is not dependent on the metric used to assess financial improvement. This examination revealed that all stationary had been violated due to the first discrepancy (Idrees and Majeed, 2022). By applying the second lag NARDL model, this research investigated the impulsive reaction among political influence and competitors’ landscape, based on the 14.78 and 0.44 tests.

The variance decomposition results are presented in Table 4 of this document. In the generalized method of moment’s calculation, utilizing the “ahead mean differential approach” eliminates the need for multiple regression because of the specific and temporal effects resulting from the committee database (Hossain and Kamrul Alam, 2017). Because both CO_2_ emissions and capital have an impact that is significant and positive, and because capital has an effect that is positive on CO_2_ emissions at a level of 1%, this indicates that there is a relationship that goes in both directions between capital and CO_2_ emissions.

## Conclusion and policy implications

This study investigates the dynamic causal relationships between financial development, renewable energy consumption and *per capita* CO_2_ emissions. We use panel co-integration methods and the panel unit root test to examine the nature of the relationships between the variables. We can conclude that, based on our findings and taking into consideration the most recent advancements in the search for alternative forms of energy in China, the tipping point, which was not reached during the research period of 1971 to 2015, may be reached in the latter stages of 2016 and 2017. We can say this with certainty because it was the subject of our investigation. Because our research focused on determining whether or not China has reached a stage where the country can make a turnaround, we did not consider a wide range of social factors in our analysis. Considering those elements and the advancements in economic policy on a global scale, additional research may be conducted on this area of the economy (Lv and Li, 2021b). These things have the potential to reveal significant truths about the natural world.

Due to China’s continuous efforts to build a national emission trading scheme, researching the potential and methods to achieve a low carbon transition are problems that are shared by all areas and businesses. These problems are especially significant for the resource-based regions, characterized by the prevalence of resource-intensive businesses that serve as the primary economic driver and the region’s pillar industries. The ongoing consolidation of global climate governance makes it more urgent for resource-based regions to transition to low-carbon economies. This shift will constitute a new barrier to the resource-based regions’ ability to achieve long-term sustainable development.

### Policy recommendation

Based on our findings, the following policy implications may be suggested:

It is recommended that the energy consumption derived from renewable sources be increased while energy derived from non-renewable sources is kept to a minimum. Implementing regulatory rules is critical in dealing with the growing CO_2_ emissions (Nabgan et al., 2017). As an alternative, public and private buildings, businesses and industries, and the electric power industry should be compelled by legislation to steadily increase the share of renewable energy sources in their energy mix shortly. It is critical to raise public awareness of renewable energy sources and clean environments to reduce emissions.

Adopting renewable energy sources and environmentally friendly technologies at every stage of the manufacturing process is critical for environmental improvement. Projects involving environmental protection methods should be encouraged to progress further.

This study investigates the impact that the growth of China’s financial system has had on carbon emissions, and draws a few conclusions and draws some policy implications as a result. It turns out that China’s financial development, and particularly the scale of its financial intermediation, is a significant contributor to the rise in carbon emissions. Therefore, when it comes to the projection of the demand for carbon emissions, it is not sufficient to simply take into account the influence of rising incomes. When formulating related policies with the goal of reducing the intensity of China’s carbon emissions, we should not only take into account the relationship adjustment that exists between changes in income level and carbon emissions. Otherwise, it is possible that the actual carbon emissions will be underestimated, making it more difficult for China to meet its target of a 40–45% reduction in the intensity of carbon emissions by the year 2020. However, the efficiency of financial intermediation does not have a large influence extent on carbon emissions, despite the fact that it may cause a change in carbon emissions. This is similar to how the influence of financial intermediation scale on carbon emissions is greater than the influence of other financial development indicators. This not only reflects the close relationship between China’s financial intermediation scale, economic growth, and carbon emissions, but it also suggests China’s future financial reform direction, and target formulation, should further emphasize the quality of financial intermediation asset use and play a positive role of financial system in the allocating efficiency of financial resources. This is because this not only reflects the close relationship between China’s financial intermediation scale, economic growth, and carbon emissions, but it also suggests China’s future financial reform direction.

The empirical findings suggest that China’s trade openness contributes to an increase in carbon emissions. The deteriorating effects that increased trade openness has had on the environment are evidence that China and other countries in the same rank have a lax regulatory framework for the environment. Therefore, in order to effectively deal with the degrading effect of globalization, policymakers in the environmental sector need to put in place and monitor a stringent regulatory framework for the environment. In addition to this benefit, renewable energy helps cut down on carbon emissions. The implication is that China ought to reduce the amount of reliance it has on fossil fuels and instead make significant investments in renewable sources of energy. Because China possesses a substantial amount of renewable energy resources that have not yet been exploited, the country’s energy portfolio ought to be expanded to include more sources of renewable power if it is to remain competitive in the face of rising carbon emissions.

### Limitations and future work

In general, there is still a lot of work that needs to be done concerning the influence of financial development and renewable energy consumption on carbon emissions in China. Some of the work that needs to be done includes a comparison of the influencing mechanism of financial development and renewable energy consumption on carbon emissions between different provinces in China, as well as a comparison of the different interactions between financial development, renewable energy consumption, and carbon emissions between China and some developed countries (including the United States, Japan, Germany, Sweden, etc.). On the basis of the vast amount of data, we can also investigate whether or not there is a relationship resembling an upside-down U between economic development and carbon emissions in China and other countries. In a nutshell, because there is only a limited dataset available, the findings presented in this paper have the potential to be significantly enriched in the near future. Nevertheless, it is hoped that the research will not only be conducive to China’s efforts to reduce the intensity of its carbon emissions, but also to China’s financial reforms.

## Data availability statement

The raw data supporting the conclusions of this article will be made available by the authors, without undue reservation.

## Author contributions

QF is responsible for the change of the research framework and the correction of the data model; JW participated in the proofreading of the language, YX was the leader of the project and the organizer and implementer of the research, SY analyzed the data, and BZ participated in the analysis of the model. All authors contributed to the article and approved the submitted version.

## Funding

This work was supported by the National Social Science Foundation of China “Research on the Formation Mechanism of Traditional Growth Mode and the Realization Path of High-quality Development” (Project No.: 21FJLB021); the project supported by the Youth Program of NSFC “Research on Growth Mechanism and Technical Change in Hybrid Economic Structure: Based on Embeded Government Competition Structure” (No.: 71703079); the General Program of Shandong NSFC “Research on the Hierarchical Measurement and Governance Mechanism of China’s Income Gap from the Dual Perspective of Government Competition and Administrative Monopoly” (No.: ZR2022MG010); and the Future Plan for Young Scholars of Shandong University.

## Conflict of interest

The authors declare that the research was conducted in the absence of any commercial or financial relationships that could be construed as a potential conflict of interest.

## Publisher’s note

All claims expressed in this article are solely those of the authors and do not necessarily represent those of their affiliated organizations, or those of the publisher, the editors and the reviewers. Any product that may be evaluated in this article, or claim that may be made by its manufacturer, is not guaranteed or endorsed by the publisher.
